# Novel Method to Quantify Trace Amounts of Isoprene and Monoterpene Secondary Organic Aerosol-Markers in Antarctic Ice

**DOI:** 10.1021/acs.est.4c09985

**Published:** 2024-11-18

**Authors:** Emilia E. Bushrod, Elizabeth R. Thomas, Alexander Zherebker, Chiara Giorio

**Affiliations:** † Yusuf Hamied Department of Chemistry, University of Cambridge, Lensfield Road, Cambridge CB2 1EW, U.K.; ‡ British Antarctic Survey, High Cross, Madingley Road, Cambridge CB3 0ET, U.K.

**Keywords:** Antarctica, biogenic, BVOC, environmental archive, ice core, Jurassic, limit of detection, mass spectrometry

## Abstract

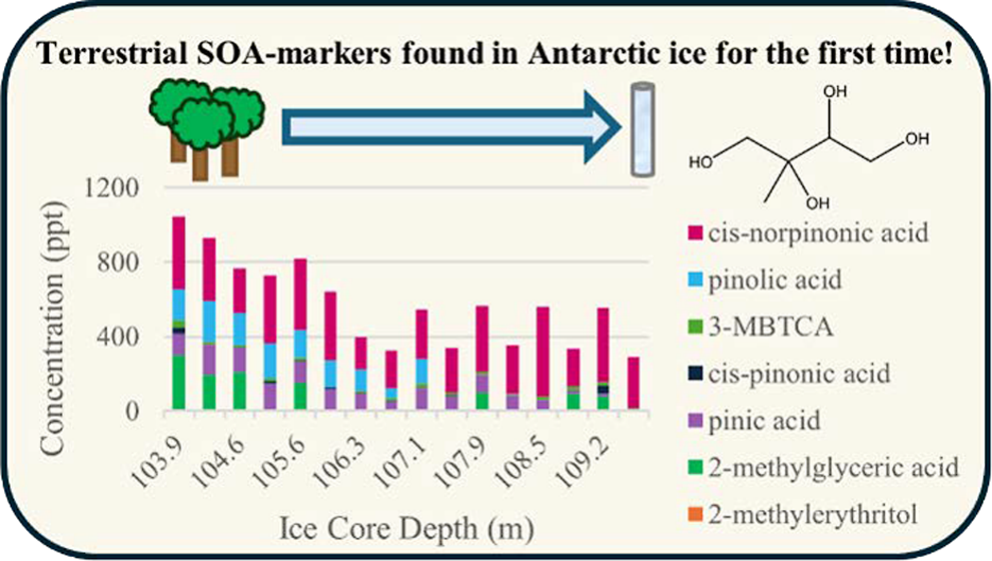

Biogenic volatile organic compounds (BVOCs) contribute to the formation of secondary organic aerosol (SOA) through atmospheric oxidation. Previously detected SOA-markers in northern hemisphere ice cores from Alaska, Greenland, Russia, and Switzerland indicate the transportation of isoprene and monoterpene oxidation products from their forestry sources to these glacial regions. Antarctica is geographically further removed from the BVOC’s source, indicating significantly lower SOA-marker concentrations are likely in southern hemisphere ice cores. The aim of this study was to develop a sensitive mass-spectrometric method to detect and quantify low-abundance SOA-markers of isoprene and monoterpenes in ice core samples. Employment of a triple quadrupole HPLC–MS method enabled limit of detections in the range of 0.4–10 ppt for nine terrestrial SOA-markers and a marker of biomass burning, levoglucosan. Quantification was conducted in the multiple reaction monitoring mode with two specific transitions monitored for each target compound. Application of the developed method onto a section of a Jurassic ice core from Antarctica revealed the presence of seven of the target compounds: 2-methylerythritol, 2-methylglyceric acid, *cis*-pinonic acid, 3-methyl-1,2,3-butanetricarboxylic acid, pinolic acid, *cis*-norpinonic acid, and pinic acid. Repeatability ranged between 2.2% and 6.2%. This is the first time that such SOA-markers have been discovered and quantified in Antarctic ice.

## Abstract 2

Minimal research exists on terrestrial organic matter in Antarctica. This novel method facilitated quantification of secondary organic aerosol markers at the trace level in an Antarctic ice core for the first time.

## Introduction

The study of non-anthropogenic organic compounds, such as secondary organic aerosol (SOA) markers, in ice is a developing field in ice core science, and as a result, there are few current records.^1,2^ The study of organic compounds in ice is vital to gain a fuller understanding of the composition of past biospheres. Volatile organic compounds (VOCs), especially isoprene and monoterpenes, are the major precursors for SOA^3,4^ on a global scale. While some algae and marine-based plant species also emit VOCs,^5^ terrestrial sources are dominant, with an estimated 90% of isoprene in the troposphere being emitted from terrestrial plants.^6^ Once released, VOCs undergo ozonolysis and other oxidation processes within the troposphere resulting in the production of specific oxidation products (i.e., SOA-markers).^7−11^ These SOA-markers can be transported for long distances and deposited in snow on glacial and polar regions.^12−15^

Due to relatively low concentrations and complexity, mass spectrometry (MS) techniques play a vital role in SOA studies.^1,16−18^ Examples of this include the use of GC–MS by Kawamura et al.^19^ who detected lipid compounds in snow samples from Greenland and Pokhrel et al.^20^ who detected oxidation products of isoprene and monoterpenes in Alaskan ice. HPLC–MS was implemented by Müller-Tautges et al.^21^ to identify carboxylic acids, including the SOA-marker pinic acid, in the Swiss Alps. Müller-Tautges et al.^21^ achieved a detection limit of 0.3 ppt for pinic acid. This method applied a pre-concentration step to increase the concentration of the analytes prior to instrumental analysis. Feltracco et al.^22^ used HPLC coupled to a triple quadrupole MS to study pinic acid and *cis*-pinonic acid (monoterpene SOA-markers) in air samples collected from Svalbard. King et al.^23^ developed a method that used HPLC–MS to detect SOA-markers in a Belukha glacier ice core (Russian Altai Mountains). In King et al.^23^’s work, several SOA-markers were detected including SOA-markers of isoprene: 2-methyltetrols (2-methylerythritol and 2-methylthreitol) and SOA-markers of monoterpenes: keto-pinic acid, *cis*-pinonic acid, and 3-methyl-1,2,3-butanetricarboxylic acid (3-MBTCA). The study tested two techniques: one implemented a pre-concentration step with less sensitive instrumentation, and one did not implement pre-concentration and used more sensitive instrumentation. King et al.^23^ achieved limits of detections (LODs) as low as 20 ppt for MBTCA and keto-pinic acid. Pokhrel et al.^20^ used a GC–MS method optimized on aerosol samples to analyze SOA-markers 2-methylerythritol, 2-methylglyceric acid, and *cis*-pinonic acid to detection limits of ≤10 ppt (exact values not stated). There are no known ice core records that investigate *cis*-norpinonic acid or nopinone.

All studies that have looked to quantify terrestrial SOA-markers in ice cores have focused on northern hemisphere ice, as they are closer to the source of precursor biogenic volatile organic compound (BVOC) emissions. However, Antarctic ice cores have the potential to extend the record over millennial time scales,^24^ with a smaller anthropogenic influence which may hamper interpretation of some northern hemisphere sites.^1,2^ Hu et al.^25^ studied isoprene and monoterpene SOA-markers in air from both the Arctic and Antarctic. The average concentration in the northern hemisphere was found to be 1 order of magnitude greater than that in the southern hemisphere. Though in smaller concentration, this indicates that a portion of the SOA-markers of isoprene and monoterpenes are reaching Antarctica and that there is a potential for them to be deposited and therefore found in the ice. Proportionally to the difference in atmospheric contents, it is possible to estimate that the concentrations of SOA-markers found in Antarctic ice cores would be in the ppt range. For example, Gambaro et al.^26^ utilized HPLC coupled with triple quadrupole MS to analyze levoglucosan, an indicator of biomass burning. Gambaro’s study reached a LOD of 3 ppt without a pre-concentration step. Up until now, no method has been presented within the literature that has quantified terrestrial SOA-markers in ice cores to such low concentrations. In this study, we present a method that enables target analysis of ten SOA-markers with all LODs being below 10 ppt.

## Materials and Methods

### Chemicals and Reagents

The list of ten standards used for method development is presented in Table 1. The target compounds represent SOA-markers of isoprene, the monoterpenes: α-pinene and β-pinene, and an indicator of biomass burning. High-purity Milli-Q water (18.2 MΩ) was prepared using a Merck Millipore Advantage A10 Water Purification System. Methanol of LC–MS grade (Fisher Scientific) was used for all of the experiments. All standards except for 3-MBTCA were commercially available as noted below. 3-MBTCA was synthesized in-house following the method from Dette et al.^27^ Synthetic procedure, purification, and NMR data can be found in the Supporting Information file. Standard analytes: 2-C-Methyl-d-erythritol (>90% (GC), Sigma-Aldrich), 2,3-dihydroxy-2-methylpropanoic acid (2-methylglyceric acid, 97%, BLD Pharm), *cis*-pinonic acid (98%, Sigma-Aldrich), 3-MBTCA (synthesized standard), (±)-pinolic acid (analytical grade, Sigma-Aldrich), *cis*-norpinonic acid (95%, enamine), (1R)-(+)-nopinone (98%, Sigma-Aldrich), (1S)-(+)-keto-pinic acid (99%, Sigma-Aldrich), pinic acid (analytical grade, Santa Cruz Biotechnology), and 1,6-anhydro-β-d-glucose (levoglucosan, 99%, Sigma-Aldrich). Individual standard solutions of 200 ppm were prepared in water with 1% methanol to aid dissolving on the day of analysis.

**Table 1:** List of Target Compounds for This Study

compound name	neutral formula	source
2-methylerythritol	C_5_H_12_O_4_	isoprene SOA-marker
2-methylglyceric acid	C_4_H_8_O_4_	isoprene SOA-marker
*cis*-pinonic acid	C_10_H_16_O_3_	α-pinene SOA-marker
3-MBTCA^a^	C_8_H_12_O_6_	α-pinene SOA-marker
pinolic acid	C_10_H_18_O_3_	α-pinene SOA-marker
*cis*-norpinonic acid	C_8_H_12_O_4_	α-pinene SOA-marker
nopinone	C_9_H_14_O_3_	β-pinene SOA-marker
keto-pinic acid	C_10_H_14_O_3_	α- and β-pinene SOA-marker
pinic acid	C_9_H_14_O_4_	α- and β-pinene SOA-marker
levoglucosan	C_6_H_10_O_5_	biomass burning

a 3-methyl-1,2,3-butanetricarboxylic acid.

A bulk standard mixture was made by combining each individual solution in water so that each analyte was in a concentration of 1 ppm. Dilutions from the bulk standard solution were made for each calibration using Milli-Q water. Sample collection tubes were precleaned by rinsing with hexane (>99.9%, HPLC, Fisher Scientific), methanol, and water.

### High-Pressure Liquid Chromatography

All analysis was performed on an ExionLC Series UHPLC with a SCIEX QTRAP 5500+ MS/MS system equipped with a Turbo V electrospray source. Chromatographic separation was achieved using a Waters XBridge C18 (3.5 μm, 3.0 mm × 150 mm) column. Separation conditions were adapted from previous works.^23,28^ Water with 0.5 mM NH_3_ and methanol with 0.5 mM NH_3_ were mobile phases A and B, respectively. The flow rate was 250 μL min^–1^. The elution gradient was as follows: 0–3 min 0% B, 3–4 min linear gradient from 0% to 30% B, 4–9 min 30% B, 9–10 min linear gradient from 30% to 100% B, 10–16 min 100% B, 16–17 min linear gradient from 100% to 0% B, 17–26 min 0% B. A post-column injection of methanol with 5 mM NH_3_ was added at a rate of 100 μL min^–1^ to increase ionization efficiency. The injection volume was 20 μL.

### Mass Spectrometry

Target profiling was conducted in multiple reaction monitoring (MRM) mode with electrospray ionization (ESI). For all analytes except for nopinone, the following parameters were used: ESI (−), 35 psi curtain gas (CUR), 9 psi collision gas (CAD), and −4500 V ion spray voltage (IS). Optimized parameters for nopinone were as follows: ESI (+), 35 psi CUR, 9 psi CAD, and 4500 V IS. An entrance potential of 10 V was set for all analytes. Ion source temperature, source gas 1, and source gas 2 were 550 °C, 50 au, and 50 au, respectively. Ion optics parameters, collision energies, and characteristic transitions are detailed in Table 2.

**Table 2:** Parameters of Target Compounds

target compound	LOD (ppt)	LOQ (ppt)	*t*_R_ (min)	repeatability (%)	repeatability concentration level (ppt)	transition type	transition (*m*/*z*)	declustering potential (V)	collision energy (V)	cell exit potential (V)
2-methylerythritol	0.4	1.3	3.51	2.6	400	quantifying	134.9→85.0	–70	–20	–9
						qualifying	134.9→103.0	–70	–14	–7
2-methylglyceric acid	4.4	14.7	2.04	6.1	500	quantifying	118.9→73.1	–10	–20	–11
						qualifying	118.9→71.1	–5	–20	–9
*cis*-pinonic acid	6.5	21.7	7.92	3.0	1	quantifying	182.9→56.9	–5	–20	–7
						qualifying	182.9→109.0	–10	–32	–11
3-MBTCA^a^	2.3	7.7	1.71	4.9	400	quantifying	203.0→185.0	–5	–18	–11
						qualifying	203.0→97.1	–5	–28	–7
pinolic acid	1.4	4.7	1.80	5.8	400	quantifying	185.0→141.1	–75	–18	–15
						qualifying	185.0→139.0	–85	–22	–17
*cis*-norpinonic acid	4.9	16.3	8.18	6.2	150	quantifying	169.0→125.0	–80	–18	–13
						qualifying	169.0→57.1	–80	–20	–5
nopinone	0.7	2.3	14.13	2.2	600	quantifying	139.0→83.0	+91	+21	+10
						qualifying	139.0→69.0	+91	+21	+10
keto-pinic acid	2.3	7.6	8.02	3.7	1	quantifying	181.0→137.1	–10	–24	–11
						qualifying	181.0→119.0	–10	–30	–11
pinic acid	3.1	10.3	1.90	2.5	450	quantifying	184.9→141.1	–85	–20	–13
						qualifying	184.9→167.2	–80	–16	–15
levoglucosan	10	33.3	3.77	3.9	1	quantifying	161.0→71.0	–70	–17	–7
						qualifying	161.0→113.0	–70	–14	–20

a 3-methyl-1,2,3-butanetricarboxylic acid. Limit of detection (LOD), limit of quantification (LOQ), and retention time (*t*_R_).

For each analyte, a quantifying and qualifying transition were used (Table 2). Quantification was done through external calibration in the range between the LOD (Table 2) and 1 ppb. The standards were analyzed in the order of lowest concentration to highest. Blanks of Milli-Q water were analyzed between each standard concentration. When ice core samples were measured, the samples were run in duplicate and with blanks of Milli-Q water between each different sample. To measure the LOD for each of the target compounds, the 1 ppm bulk standard solution of all the analytes in water was further diluted to 1 ppb, 100 ppt, 50 ppt, 20 ppt, 10 ppt, 5 ppt, and 1 ppt with water. These standards were analyzed in triplicate using the full method described above.

### Data Processing

Data was acquired using Sciex Analyst 1.7 and saved as *.wiff files. Peak detection and integration were performed using SCIEX OS 3.2. All recovered peaks were manually reviewed, and peak integration was adjusted where appropriate. To perform calibration and calculate the concentrations, the raw peak area data were exported to Microsoft Excel (Office 365). Linear calibration was used for all analytes using a linear least-squares regression.

Calculation of the LOD for each target compound was performed using the Hubaux and Vos^29^ method in OriginPro v10. The LOQ for each of the target compounds was calculated using the formula below
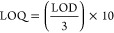


The linearity determination coefficient (*R*^2^) value was calculated and used to identify the linearity range for each target compound (*R*^2^ > 0.9, *p*-value >0.05). Repeatability measurements were taken for each target compound to test the variability between repeated injections across multiple instrumental runs, following Worsfold et al.^30^ Calibration standards were injected in triplicate for each LOD test run and in singlet at the beginning of each instrumental run where samples were measured. Additionally, quality check standards were repeated at varying times throughout runs to assess degradation and monitor the instrument response. The matrix effects were evaluated across the full calibration concentration range between two otherwise identical standards, the first made up in Milli-Q water and the second in melted ice core. The matrix effect was determined using both *t*-test statistical analysis and percentage differences between slopes. Concentrations are presented in parts per trillion. Additional information on repeatability and matrix effects, including full methods, can be found in the Supporting Information.

### Ice Core Sample Collection

The Jurassic ice core was drilled on the Antarctic Peninsula (74.33°S, 73.06°W) in 2012.^31^ The age scale (1874–2011 C.E.) was derived using non-sea-salt sulfate ([nssSO_4_^2–^]) and hydrogen peroxide (H_2_O_2_) verified using volcanic reference horizons.^32,33^ The ice core was prepared and melted in a cold room at The British Antarctic Survey and fractions from approximately 4.25 cm were collected for this research. Ice core fractions of approximately 6 mL were melted and collected in glass tubes during a continuous flow analysis campaign using a BÜCHI C-660 fraction collector. Once collected, the tubes were sealed using parafilm and stored in a −25 °C freezer.

A total of 16 samples were collected along with blank Milli-Q water that had been treated in the same way as the ice core samples. Before analysis, samples were melted in a class-100 clean room at room temperature, shaken, and decanted into 1.5 mL Shimadzu polypropylene vials. The vials were sealed by Shimadzu red silicon caps with a polytetrafluoroethylene septum suitable for the chromatographic system autosampler.

## Results and Discussion

### Instrument Optimization

The final list of ion transitions and instrument parameters for MRM experiments is presented in Table 2. Ionization and MS parameters were systematically and manually optimized for each compound individually by injection of 10 ppb solutions in water. As part of optimization, the mass spectrometer’s automatic optimization setting was utilized to select the four most viable (as suggested by the system) product ions for each analyte during direct injection. In all cases, the list of product ions was confirmed by manual optimization by consecutive LC–MS experiments. The transitions were tested through the HPLC-MS system using the MRM method, and for each analyte, the two most intense MRM transitions were chosen for quantification and for qualitative control (Figure S1). If a compound’s greatest intensity transition(s) were different between direct injection and HPLC–MS (MRM), the two most intense transitions in the HPLC (MRM) were chosen for quantifying and qualitative control (Figure S2). Suggestions of fragmentation are provided in the Supporting Information, but further research would be required to prove the relevant fragmentation structures.

For 2-methylerythritol, 2-methylglyceric acid, and 3-MBTCA, the most representative transitions in MRM were 135 → 85, 119 → 73, and 203 → 185. Both pinic acid and *cis*-pinonic acid have been analyzed by HPLC–MS by Feltracco et al.^34^ which used a quantifying transition of 185 → 141 for pinic and 183 → 139 for *cis*-pinonic acid. In our work, the same quantifying transition was used for pinic acid, but for *cis*-pinonic acid, the 183 → 57 transition was used instead. This transition was also observed in the MRM method used by Witowski et al.^35^ For levoglucosan, both quantitative and qualitative transitions have been used in the previous MRM method by Gambaro et al.^26^ Chosen transitions for pinolic acid, *cis*-norpinonic acid, nopinone, and keto-pinic acid are shown in Table 2. To our knowledge, MRM methods have not previously been applied to detect and quantify these molecules. In all cases, qualitative transitions were also chosen for quality control. An example of an extracted-ion chromatogram (XIC) for both quantitative and qualitative transitions can be seen in Figure S3. Calibration curves over the full concentration range (1 ppt–1 ppb) for sample analysis can be seen in Figure S4. Calibration curves for all detected compounds show linearity with *R*^2^ > 0.978 and *p*-value ≥ 0.685.

### Method Validation

The LODs and LOQs for each of the ten target compounds were established and can be viewed in Table 2. All of the LODs achieved in this study are ≤10 ppt. The two lowest LOD values were obtained for 2-methylerythritol and nopinone at 0.4 and 0.7 ppt, respectively. The lowest recorded LODs in other ice core studies for 2-methylerythritol, 2-methylglyceric acid, and *cis*-pinonic acid are listed as ≤10 ppt^20,36^ and for 3-MBTCA, pinolic acid, and keto-pinic are 20, 590, and 20 ppt, respectively.^23,28^ The results presented in our study are at least equivalent in magnitude and several orders of magnitude improved in most cases. For pinic acid and levoglucosan, previous studies have achieved LODs of 1 order of magnitude lower than our method.^21,26^ Previously mentioned non-ice core MRM methods^34,37^ have attained LODs of 1.2 and 1.6 ppt for pinic acid and *cis*-pinonic acid, respectively, and 32, 8, and 4 ppb for 2-methylerythritol, 2-methylglyceric acid, and 3-MBTCA, respectively. Our study achieves LODs in the same order of magnitude for both pinic and pinonic acid and LODs of at least 2 orders of magnitude lower for 2-methylerythritol, 2-methylglyceric acid, and 3-MBTCA. There are no known investigations into *cis*-norpinonic acid or nopinone in ice cores, but non-ice core studies have achieved LODs of 1130 and ≤10 ppt, respectively,^38,39^ which are up to 4 orders of magnitude larger than the LODs of the method presented here.

**Figure 1:**
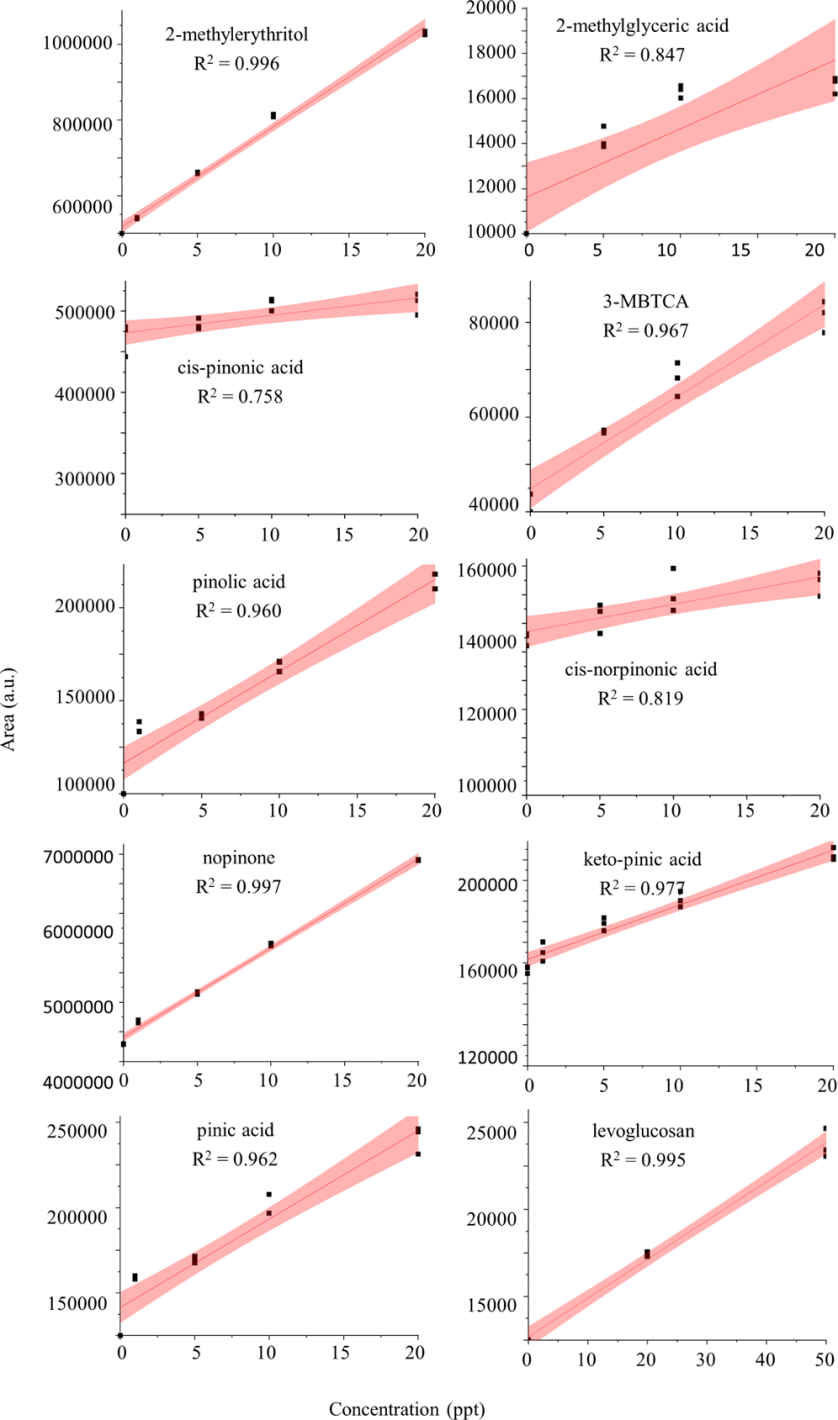
Scatterplots of the produced standard concentration versus the recorded area of each of the target compounds. The determination coefficient (*R*^2^) value and trendline are shown on each plot. The pink error band represents a 95% confidence interval.

All nine terrestrial SOA-marker compounds tested were found to exhibit linearity in the target LOD range (1–20 ppt) of *R*^2^ > 0.75 as reported in Figure 1. Levoglucosan was found to exhibit linearity at its target LOD range (1–50 ppt) of *R*^2^ = 0.99. All instrumental repeatability values are <6.2% at the point of plateau (Figure S5 and Table 2) indicating acceptable instrumental stability. Full repeatability detail, including the repeatability plots for each target compound, can be found in the Supporting Information (Figure S5). Target compound degradation at room temperature was tested, and results suggest that a degradation correction would need to be considered if using this method on a larger scale due to the increased time of HPLC–MS batches being at room temperature (Figure S6). Due to the small number of samples analyzed in this study, we have not included a degradation correction; however, for larger batch analysis, correction would be required.

The compounds that are most greatly affected by degradation over longer time periods are 2-methylerythritol, 2-methylglyceric acid, pinic acid, 3-MBTCA, and nopinone. The matrix effect was assessed, and results are displayed in Table 3 with plots shown in Figure S7. The maximum difference between the slopes of the water standards and of the ice standards is <15% for all target compounds, excluding 3-MBTCA, indicating a minimal matrix effect. 3-MBTCA has a difference of 32.1% suggesting that this compound could be affected by the matrix. The results of the *t*-test show all compounds exhibit a p value of higher than 0.05, which means that the two slopes are equal within a 95% confidence level.

**Table 3:** Results of the Matrix Effect Assessment across the Full Calibration Concentration Range (0–1000 ppt)^a^

compound	slope		difference of slopes (%)	*t*-test p-value
	water	ice		
2-methylerythritol	70952.4 (±182.7)	62677.8 (±243.6)	12.4 (±0.6)	0.863
2-methylglyceric acid	7545.8 (±66.9)	6634.9 (±55.7)	12.8 (±1.7)	0.908
*cis*-pinonic acid	14823.8 (±122.4)	13125.5 (±94.0)	12.2 (±1.6)	0.688
3-MBTCA	34763.8 (±406.0)	25141.1 (±406.0)	32.1 (±2.7)	0.685
pinolic acid	69528.5 (±303.8)	66812.7 (±336.3)	3.8 (±0.9)	0.977
*cis*-norpinonic acid	10030.1 (±123.0)	8652.0 (±107.0)	14.8 (±2.5)	0.972
nopinone	844093.0 (±16791.3)	768207.2 (±8668.1)	9.4 (±3.2)	0.885
keto-pinic acid	74863.9 (±272.1)	66452.4 (±363.8)	11.9 (±0.9)	0.870
pinic acid	77545.6 (±305.7)	74527.98 (±356.3)	4.0 (±0.9)	0.976
levoglucosan	720.3 (±5.0)	706.6 (±5.3)	2.0 (±1.4)	0.834

a The slopes of both sets of standards are shown with their respective uncertainties in brackets. The percentage difference between the slopes is shown with its respective uncertainties. The *p* values of the *t*-test are shown where α was set to 0.05.

### Detection of SOA-markers

Samples taken from a 4.25 cm section of the Jurassic ice core were analyzed using the method detailed in this study. The age scale and relationship between depth and age of the Jurassic ice core was deduced and described by Emanuelsson et al.^31^ using annual layer counting. They confirmed the accuracy of the dating by evaluating results against known volcanic data.^32,33^

Of the ten target compounds, seven were found to be present within the section of ice. They were SOA-markers of isoprene: 2-methylerythritol and 2-methylglyceric acid and SOA-markers of monoterpenes: *cis*-pinonic acid, 3-MBTCA, pinolic acid, *cis*-norpinonic acid, and pinic acid.

**Figure 2:**
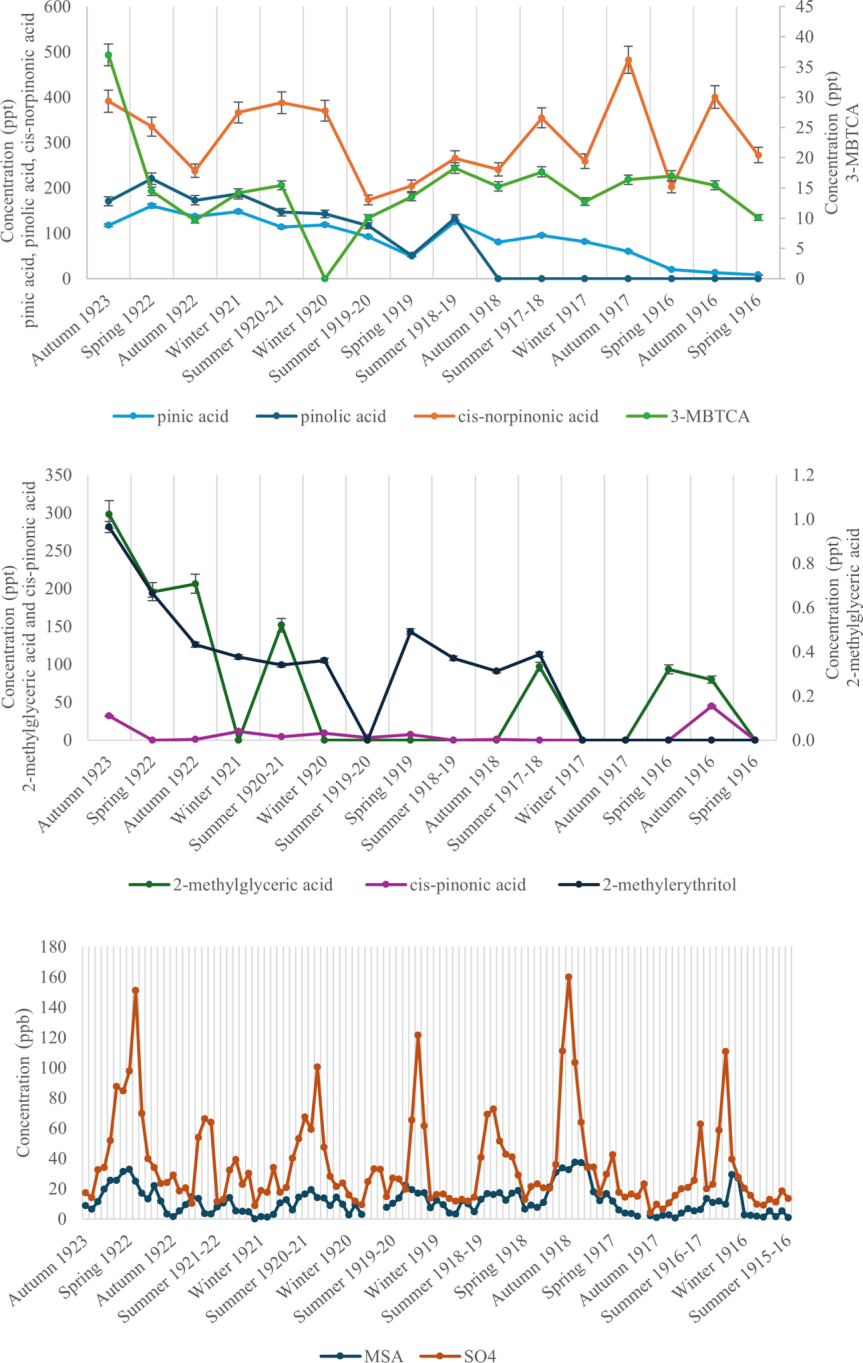
Individual Jurassic sample concentrations. SOA-markers that show similar trends: pinic acid pinolic acid, *cis*-norpinonic acid, and 3-MBTCA (top). SOA-markers that do not show trends: 2-methylglyceric acid, *cis*-pinonic acid, and 2-methylerythritol (middle). DMS-markers that peak during the spring/summer: methanesulfonic acid (MSA) and sulfate ion (SO_4_^2–^) (bottom). Error bars are respective repeatability values (Table 2) as percentages.

Compounds were detected at concentrations ranging from 0.4 to 482 ppt. The isoprene SOA-marker 2-methylerythritol was detected at the lowest concentrations, with the α-pinene SOA-marker *cis*-norpinonic acid detected at the highest concentrations. Dating of the ice core suggests that the section analyzed here ranges from the years 1916 to 1923.^31^ To obtain annual average concentrations within each year, a mean average was calculated for concentrations exceeding the LOD. The final values are displayed in Table 4. The concentrations of the individual samples can be seen plotted in Figure 2.

**Table 4:** Annual Average Concentrations of Each of the Seven Detected SOA-Markers and Two DMS-Markers^a^

year	sample depth range (m)	annual average concentration								
		(ppt)							(ppb)	
		2-methylerythritol	2-methylglyceric acid	pinic acid	*cis*-pinonic acid	3-MBTCA	pinolic acid	*cis*-norpinonic acid	MSA	SO_4_^2–^
1916	109.53–108.86	-	57.6	14.0	14.9	14.2	-	291.4	8.3	29.8
1917	108.53–108.19	-	-	71.1	-	14.5	-	370.5	5.3	17.4
1918	107.86–107.53	-	48.4	88.2	-	16.4	-	297.4	21.5	50.3
1919	107.05–106.57	0.4	-	87.6	-	15.9	92.3	234.9	12.2	37.4
1920	106.26–105.64	-	50.5	108.4	-	8.5	135.6	310.5	10.9	33.9
1921	105.14	-	-	147.6	11.6	14.2	186.7	366.3	6.4	30.5
1922	104.64–104.29	0.5	201.0	148.7	-	12.0	196.6	286.3	16.7	53.0
1923	103.94	1.0	297.6	117.3	31.8	37.0	170.3	391.2	11.5	24.3

a The “-” indicates that the average could not be calculated due to concentrations being below LODs.

Levoglucosan, a marker for biomass burning that has been detected in previous Antarctic ice cores, was not observed in our samples. Significant biomass burning did occur in the early 1900s, so we would expect levoglucosan to have been emitted during the observed time period. Reasoning for this could be that the transport and deposition of atmospheric particles such as levoglucosan is less efficient in interglacial periods compared to glacial periods, as described in Gambaro et al.^26^ Due to levoglucosan spiking more sporadically than SOA-markers that are almost constantly released, our small data set that covers a short time frame could be a period without large impact from biomass burning. Analysis of a greater amount of ice infers this further.

Though no previous investigation has detected these compounds in Antarctic ice, aerosol studies suggested that concentrations would be on average 1 order of magnitude lower than equivalent compounds detected in northern hemisphere ice.^25^ Comparison with northern hemisphere ice core studies reveals that the concentrations we detected of 2-methylerythritol, *cis*-pinonic acid, and pinic acid in the Antarctic ice core were 1–3 orders of magnitude smaller than detected for the same time scale in an Alaskan ice core (Pokhrel et al.^20^ found 2-methylerythritol ranging from 107 to 1113 ppt, *cis*-pinonic acid from 80 to 161 ppt, and pinic acid from 135 to 225 ppt between 1918 and 1923). However, pinic acid was detected at only sub-ppt concentrations by Müller-Tautges et al.^21^ in an ice core from the Swiss Alps for the same date range. Though keto-pinic acid, *cis*-pinonic acid, and MBTCA were reported to be detected from within a Russian ice core, the authors did not present their concentrations.^23^

Pinic acid, 3-MBTCA, and pinolic acid showed an overall increase in the annual concentration from 1916 to 1923. Calculation of the annual concentrations of methanesulfonic acid (MSA) and a sulfate ion (SO_4_^2–^) measured for the same depth range^31^ showed a lack of correlation with the SOA-markers. Taking that MSA and the sulfate ion are the oxidation products of dimethylsulfide, produced by marine algae during austral spring and summer phytoplankton bloom,^33,40^ this likely indicates the terrestrial origin of detected SOA-markers in the ice core.

The time series for SOA-marker compounds provided more insight compared to the annual values. Despite a limited data set, it was possible to resolve spring/summer and autumn/winter seasons in the time series for some of the compounds as shown in Figure 2. It is clearly seen that intrayear variation is substantial for all the compounds. For example, for *cis*-norpinonic acid, the concentration in spring/summer is 2-fold higher compared to autumn/winter. Pinic acid, pinolic acid, *cis*-norpinonic acid, and 3-MBTCA all show similar trends, with spikes often observed in spring/summer and troughs in autumn/winter. The remaining detected compounds, 2-methylglyceric acid, *cis*-pinonic acid, and 2-methylerythritol, do not possess enough information in these data points to positively identify a trend. Both MSA and the sulfate ion demonstrated a clear seasonal trend. Unlike the correlation observed in Cui et al.,^41^ comparison of our annual maximum values for SOA- and DMS-markers showed a lack of correlation, supporting the terrestrial origin of detected organic compounds. Stohl and Sodemann^42^ show that the majority of Antarctic air originates from ocean sources but that some comes from the other southern hemisphere continents, South America, South Africa, and Australia. Air transport from these continents has a significantly greater contribution on longer time scales due to the long-range transport. They note that more transport from these continents reaches Antarctica during southern hemisphere winter (June–August) than summer (December–February) but that they were unsure if the time scale allowed for aerosols to reach Antarctica. Back trajectories by Thomas et al.^43^ from the Jurassic region between 1979 and 2010 support these transport sources with ∼51% of 5 day back trajectory air originating from the Amundsen Bellingshausen Sea (65–180°W). Given this, we would expect terrestrial sources to include South America (Patagonia) and Australia. Given the predominant terrestrial source of our SOA-markers, our results confirm that long-range transport of aerosols emitted from these continents can reach Antarctica. Some compounds show spikes in spring/summer but some in autumn/winter in the short time scale investigated. These varying trends could be the result of a balance between greater terrestrial BVOC precursor emissions during the summer, in contrast with reduced air transport from other continents during this time. A longer ice core section with a higher resolution (e.g., collecting fractions as small as 0.2 mL in volume each compared to the 3 mL used in this study) would need to be analyzed to confirm any observed trends, but we can confirm that this is the first study to present detection and quantification of all seven of these SOA-marker compounds in Antarctic ice. These results are encouraging and suggest that further analysis of a larger section of this ice core and other Antarctic ice cores is possible and that these data would provide more information on terrestrial biogenic sources reaching Antarctica.

Previous investigations^42^ suggest that the source of Antarctic air is dominated by meridional transport from within or immediately surrounding the continent with limited input from other continents. Due to the predominant terrestrial source of the compounds analyzed in our study, finding these analytes in Antarctica demonstrates the long-range transportation from forest regions. The summation of the total concentrations of all isoprene-derived SOA-markers (2-methylerythritol and 2-methylglyceric acid) across all samples recorded in this study is approximately seven times smaller than the summation of the total concentrations of all pinene-derived SOA-markers (pinic acid, *cis*-pinonic acid, 3-MBTCA, pinolic acid, and *cis*-norpinonic acid) across all samples recorded in this study recorded at 1125.4 and 8056.6 ppt, respectively. Investigations have shown that pinenes dominate the SOA production in the lower troposphere, but isoprene dominates in the upper (free) troposphere.^44^ We would expect, therefore, isoprene SOA-markers to have a longer range of atmospheric transportation, but in fact, we see significantly greater pinene-derived SOA-marker concentration in this section of the Antarctic ice core than isoprene-derived. The broader environmental implications of detecting SOA-markers in Antarctica involve unlocking the biogenic archive within older Antarctic ice to infer changes within the environment. SOA-markers and their BVOC precursors are intrinsically linked to production of tropospheric ozone which in part drives global climate through oxidation of trace gases.^10^ Through analysis of SOA-markers in Antarctic ice, we have the potential to implicate changes in BVOCs which are indicators of land use change.^45^ Biosphere reconstruction on an Antarctic ice core scale through analysis of SOA-markers has the potential to allow assessments of past changes in the climate and environment while encountering less anthropogenic contribution than northern hemisphere ice cores.

The production of this novel method gives confidence that quantitative analysis of terrestrial organic compounds in ice cores in Antarctica is possible without the requirement of a pre-concentration step prior to analysis. This method opens the field to the exploration of terrestrial biogenic sources in Antarctic ice, maintaining the best possible time resolution in the environmental archive.

## Supporting Information Available

Additional experimental methods, including the synthesis of 3-MBTCA, repeatability, and matrix effect assessment, method optimization details, tentative fragmentation suggestions, MRM spectra for all target compounds, and plots used for calibration, repeatability, and degradation (PDF)

## Abbreviations

BVOC: biogenic volatile organic compound
SOA: secondary organic aerosol
LOD: limit of detection
MS: mass spectrometry
GC–MS: gas chromatography–mass spectrometry
HPLC–MS: high-performance liquid chromatography–mass spectrometry
3-MBTCA: 3-methyl-1,2-3-butanetricarboxylic acid
MRM: multiple reaction monitoring
ESI: electrospray ionization
CUR: curtain gas
CAD: collision gas
IS: ion spray
LOQ: limit of quantification
CE: common era
CFA: continuous flow analysis
DMS: dimethylsulfide
