# Supplementary material for: Novel Method to Quantify Trace Amounts of Isoprene and Monoterpene Secondary Organic Aerosol-Markers in Antarctic Ice
Source: Environ Sci Technol. 2024 Nov 18;58(48):21177–85. doi: 10.1021/acs.est.4c09985 (PMC11618991; doi:10.1021/acs.est.4c09985)
Supplement: Supplementary file 1 — es4c09985_si_001.pdf [file es4c09985_si_001.pdf]

Additional experimental methods and method optimisation plots

A Novel Method to Quantify Trace Amounts of Isoprene and Monoterpene Secondary Organic Aerosol Markers in Antarctic Ice

Emilia E. Bushrod*[1,2], Elizabeth R. Thomas[2], Alexander Zherebker[1], Chiara Giorio[1*].

1. Yusuf Hamied Department of Chemistry, University of Cambridge, Lensfield Road, Cambridge, CB2 1EW.
2. British Antarctic Survey, High Cross, Madlingley Road, Cambridge, CB3 0ET.

Summary: 14 pages, 1 table, 7 figures.

Synthesis of 3-methylbutane-1,2,3-tricarboxylic acid (3-MBTCA)

Step 1: synthesis of 3-methylbutane-1,2,3-tricarboxylate

Under a nitrogen balloon, a solution of n-butyllithium in hexanes (1.6 M, 9 mL, 14.4 mmol) was added to a solution of $^{i}Pr_2NH$ (di-isopropyl amine, 2.1 mL, 15 mmol) in commercial anhydrous THF (tetrahydrofuran, 10 mL) at 0 °C under ice-bath cooling. The solution was stirred at 0 °C for 80 minutes. The solution was cooled to -70 °C with dry ice, then a solution of methyl 2-methylpropanoate (1.47 g, 14.4 mmol) in anhydrous THF (5 mL) was added over a period of 75 minutes. The solution was stirred for a further 15 minutes. A solution of dimethyl maleate (2.08 g, 14.4 mmol) in anhydrous THF (5 mL) was added over 60 minutes. The colour of the solution changed from colourless to yellow. The solution was stirred at -70 °C for a further 30 minutes before being poured into $HCl_{(aq)}$ (1 M, 30 mL) under ice bath cooling. The phases were extracted with Et_2O (2 x 20 mL, 1 x 15 mL) and all organic phases combined. Solvents were removed using rotary evaporation (40 °C, reduced pressure) to obtain 3-methylbutane-1,2,3-tricarboxylate as a colourless oil.

Step 2: synthesis of 3-methylbutane-1,2,3-tricarboxylic acid (3-MBTCA)

3-methylbutane-1,2,3-tricarboxylate (1.95 g, 7.9 mmol) was added to a solution of NaOH (5.01 g, 0.125 mol) in H_2O (15 mL) and methanol (15 mL). The solution was heated to reflux for 4 hours. While cooling in an ice bath, the solution was acidified with HCl (1 M, 14 mL) to pH 1 to form a colourless solid. The suspension was extracted with Et_2O (7 x 17 mL) then all organic phases were combined and dried with $MgSO_4$. The solvents were removed with rotary evaporation (40 °C, reduced pressure) to give a solid colourless residue. The residue and NaOH (1.03 g, 0.026 mol) was added to water (dissolve) and heated at reflux for 2 hours. The solution was cooled in an ice bath and brought to pH 1 with HCl (1 M, 6.0 mL). The aqueous and organic phases were separated and extracted with Et_2O (8 x 9 mL). Organic phases were combined, and solvent was removed using rotary evaporation (40 °C, reduced pressure) and finally a high vacuum pump (room temperature, ca. 0.05bar) to give 3-MBTCA as a colourless solid (1.61 g, 50%). ^{1}H NMR (500 MHz, D_2O): δ = 3.17 (dd, ^{3}J = 3.7Hz, 3.6Hz, 1 H, CH), 2.71 (dd, ^{2}J = 11.5Hz, ^{3}J =11.4Hz, 1 H, 1 H of CH_2), 2.56 (dd, ^{2}J = 3.7 Hz, ^{3}J = 3.6Hz, 1 H, 1 H of CH_2), 1.15 and 1.13 (2 s, 3 H each, CH_3).

Assessment of the repeatability of the method

The peak areas of each of the 2 or 3 injections at each sample or standard concentration respectively were taken (standard concentrations were from 3 different runs on 3 different days, and the sample concentrations were from the Jurassic ice core results), and the standard deviation determined. The difference between the areas was divided by the mean average of each of the 3 injections and multiplied by 100 to give a percentage difference. This was repeated at all standard concentrations (1 ppt – 1000 ppt) and sample values. A total mean average at each concentration was calculated and fitted with an exponential trendline. The percentage difference at which the trendline is at a plateau is taken as repeatability (%). This was done for each of the target compounds. The plots used to calculate repeatability for each compound can be found in Fig S5.

Assessment of the matrix effects of the method

Two standard sets at each concentration (1-1000 ppt) were prepared containing all target compounds; (1) in MilliQ water as described in the method of the main paper, and (2) in melted ice core. Apart from their base solvent, the two standards are identical in composition. The standards were run sequentially from lowest concentration to highest concentration with MilliQ blanks between triplicate injections, e.g. triplicate 1ppt in water, followed by a blank, followed by triplicate 1ppt in ice, followed by a blank, etc.. Blank ice core was also analysed with blank MilliQ water between injections. The areas of each set of standards of each target compound were plotted against concentration (Fig. S7). Linear trendlines were fitted and slopes calculated. The percentage difference between the slopes of water standards and ice standards of each separate target compound were established with uncertainty calculated using Microsoft Excel's LINEST array function. The error of the uncertainty was calculated using error propagation through dividing the summation of the uncertainties of both slopes by the mean average of both slopes and multiplying by 100 to make a percentage. A t-test was performed on Microsoft Excel using "t-Test: Two-Sample Assuming Equal Variances" function between the water standards and the ice standards of each target compound with the level of significance set to 0.05. The recorded p-value should be >0.05 to be classified as significant.

Tentative Fragmentation Suggestions

Target Compound	Transition Type	Transition (m/z)	Suggested Fragment Formula
2-methylerythritol	quantifying	134.9→85.0	$[CH_2CHCH_2C(CH_3)OH]^-$
	qualifying	134.9→103.0	$[C(CH_3)_2(OH)CH(OH)CH_2]^-$
2-methylglyceric acid	quantifying	118.9→73.1	$[CH_2CH_2COOH]^-$
	qualifying	118.9→71.1	$[CH_2C(CH_2)CH_2OH]^-$
cis-pinonic acid	quantifying	182.9→56.9	$[CH_3COCH_2]^-$
	qualifying	182.9→109.0	$[CH(CH_3)_2CH_2C(CH_2)CCH_2]^-$
3-MBTCA*	quantifying	203.0→185.0	$COOHC(CH_3)_2C(COOH)CHCO]^-$
	qualifying	203.0→97.1	$[CHCHCHCHCOOH]^-$
pinolic acid	quantifying	185.0→141.1	$[C_4H_4(CH_3)_2CH_2COOH]^-$
	qualifying	185.0→139.0	$[CH_2C_4H_4(CH_3)_2C(O)CH_3]^-$
cis-norpinonic acid	quantifying	169.0→125.0	$[C_4H_4(CH_3)_2C(O)CH_3]^-$
	qualifying	169.0→57.1	$[CH_3C(O)CH_2]^-$
nopinone	quantifying	139.0→83.0	$[CH_2CH_2CHCHCHO]^-$
	qualifying	139.0→69.0	$[CH_2CHCHCHO]^-$
(1S)-keto-pinic acid	quantifying	181.0→137.1	$[C_6H_7(O)C(CH_3)_2]^-$
	qualifying	181.0→119.0	$[C_6H_5C(CH_3)_2]^-$
pinic acid	quantifying	184.9→141.1	$[C_4H_4(CH_3)_2CH_2COOH]^-$
	qualifying	184.9→167.2	$[COOHC_4H_3(CH_3)_2CHC(O)]^-$
levoglucosan	quantifying	161.0→71.0	$[C_3H_3O_2]^-$
	qualifying	161.0→113.0	$[C_6H_9O(O)]^-$

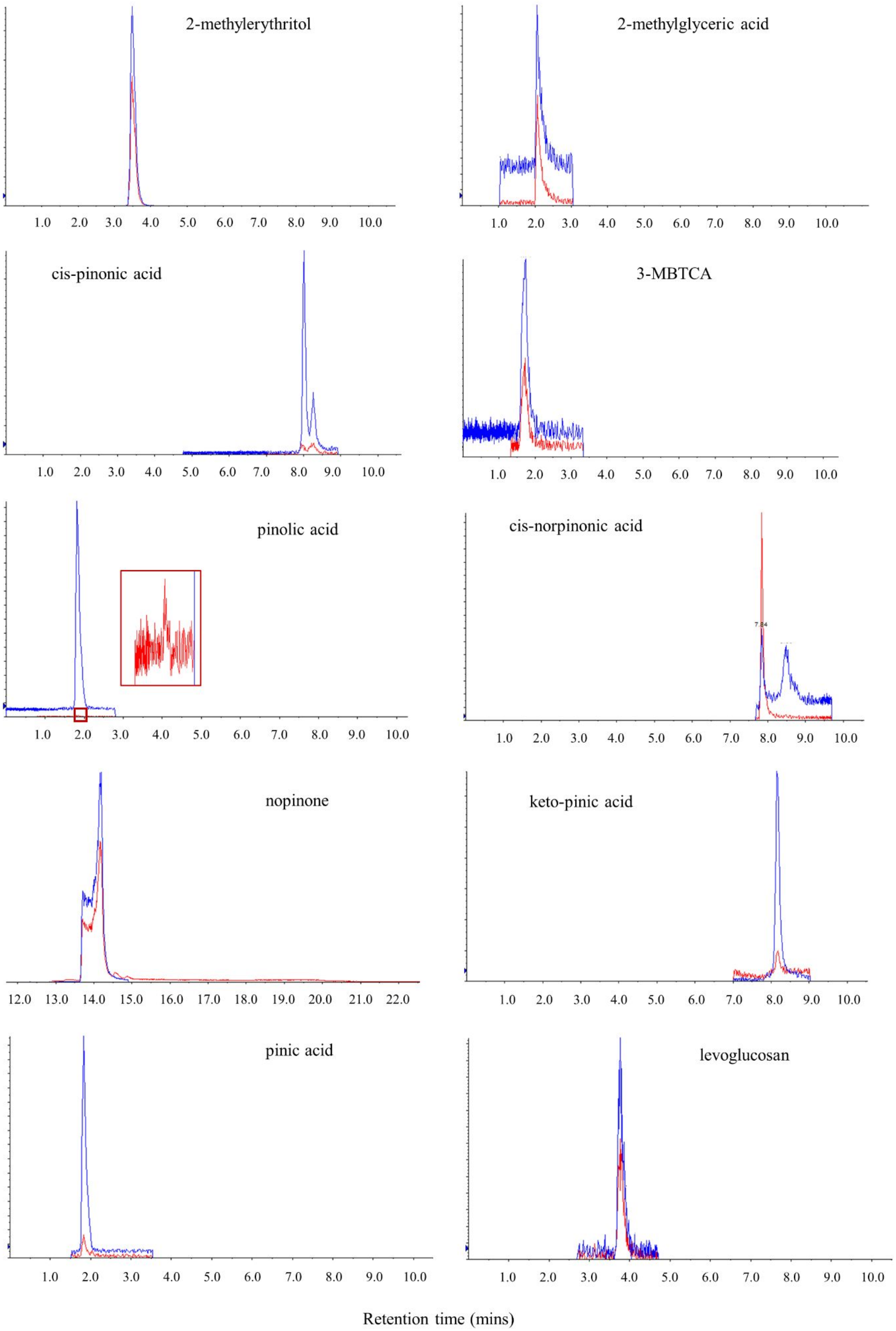

Figure S1. MRM spectra for each target compound at 100 ppt. Quantifying transitions are shown in blue, qualifying transitions are shown in red.

Figure S2 (a):

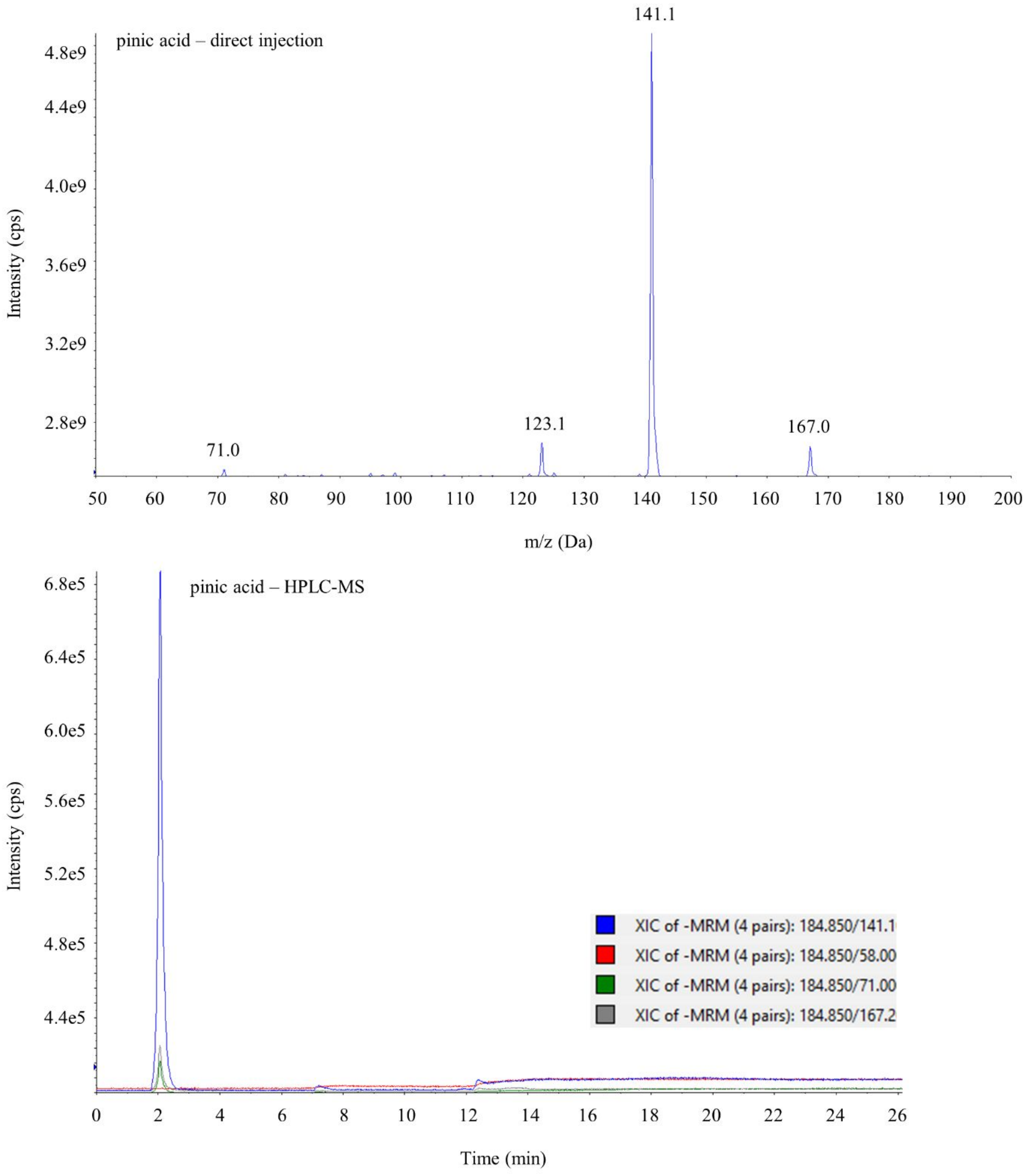

pinic acid – direct injection
141.1
123.1
167.0
71.0
4.8e9
4.4e9
4.0e9
3.6e9
3.2e9
2.8e9
Intensity (cps)
50 60 70 80 90 100 110 120 130 140 150 160 170 180 190 200
m/z (Da)
pinic acid – HPLC-MS
6.8e5
6.4e5
6.0e5
5.6e5
5.2e5
4.8e5
4.4e5
Intensity (cps)
XIC of -MRM (4 pairs): 184.850/141.1
XIC of -MRM (4 pairs): 184.850/58.00
XIC of -MRM (4 pairs): 184.850/71.00
XIC of -MRM (4 pairs): 184.850/167.2
0 2 4 6 8 10 12 14 16 18 20 22 24 26
Time (min)

Figure S2 (b):

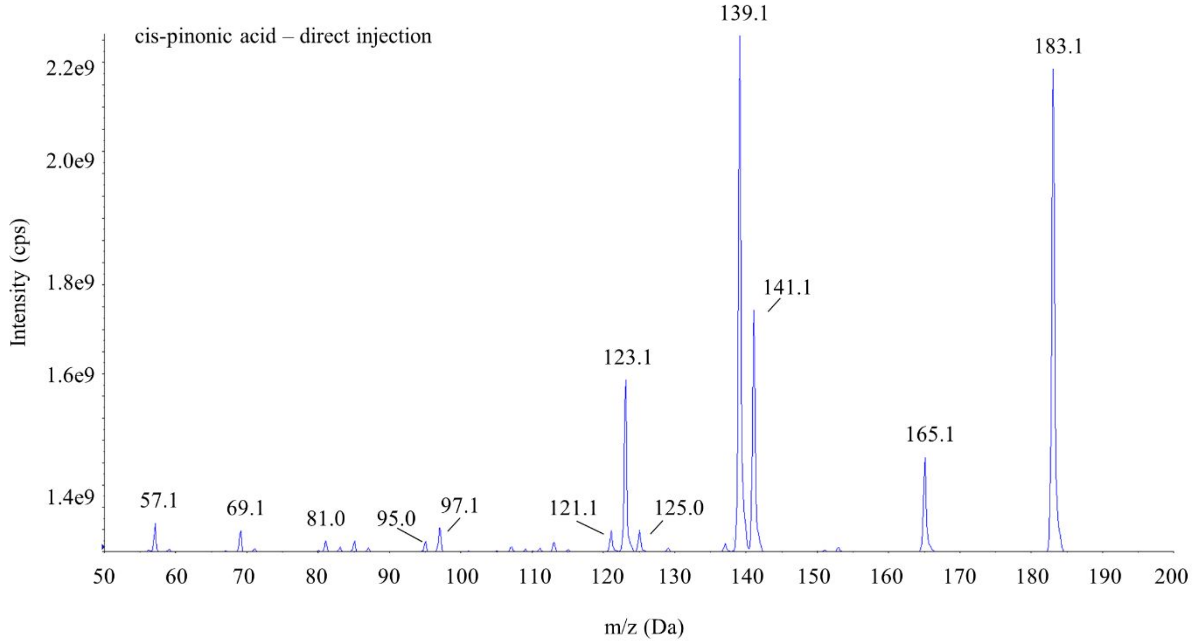

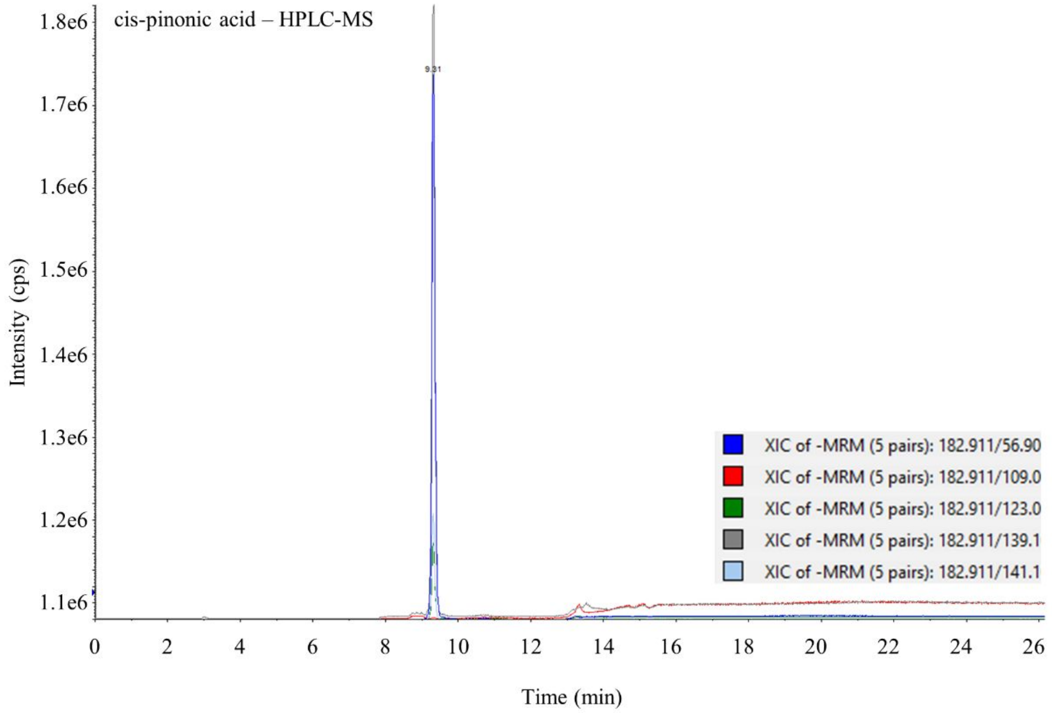

Figure S2. Examples of plots used for transition selecting where (a) direct injection and HPLC-MS (MRM) produce the same two transitions with the highest intensity (example: pinic acid), and (b) direct injection and HPLC-MS produce different transitions with the highest intensity (example: cis-pinonic acid). For all compounds that align with (b), the two transitions with the greatest intensity at HPLC-MS (MRM) were chosen as quantitative and qualitative transitions. The direct injection plots are Q3 experiments of individual standards at 10 ppb during manual optimization, the HPLC-MS plots are XIC experiments of individual standards at 10ppb.

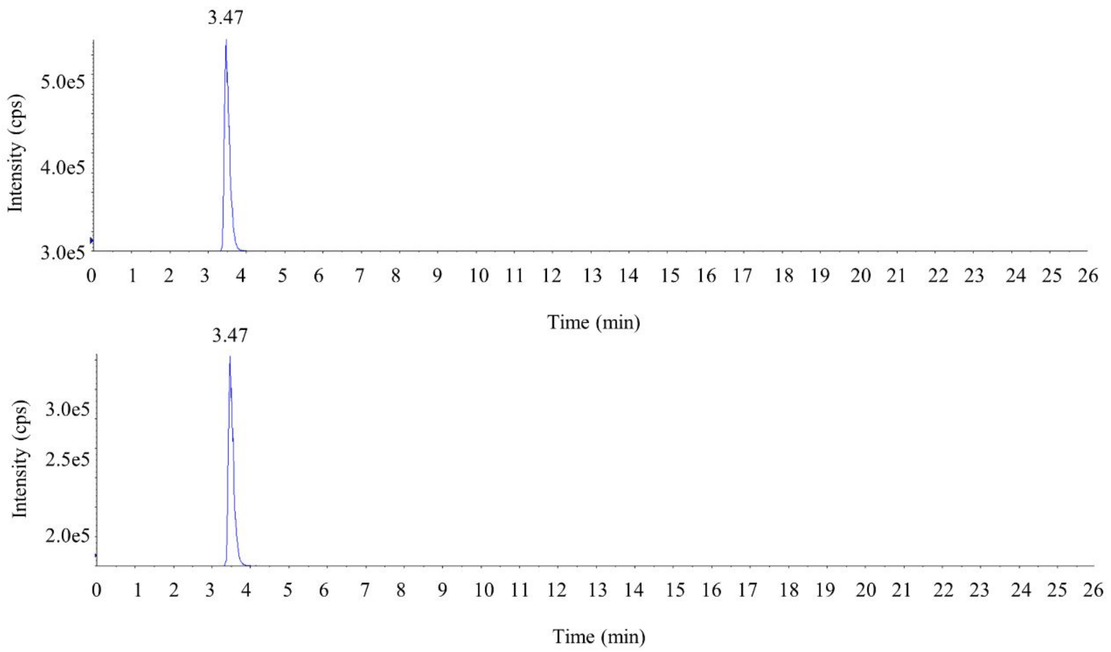

Figure S3. Example XIC of 2-methylerythritols quantitative (top) and qualitative (bottom) transitions from a full standard MRM at 100 ppt.

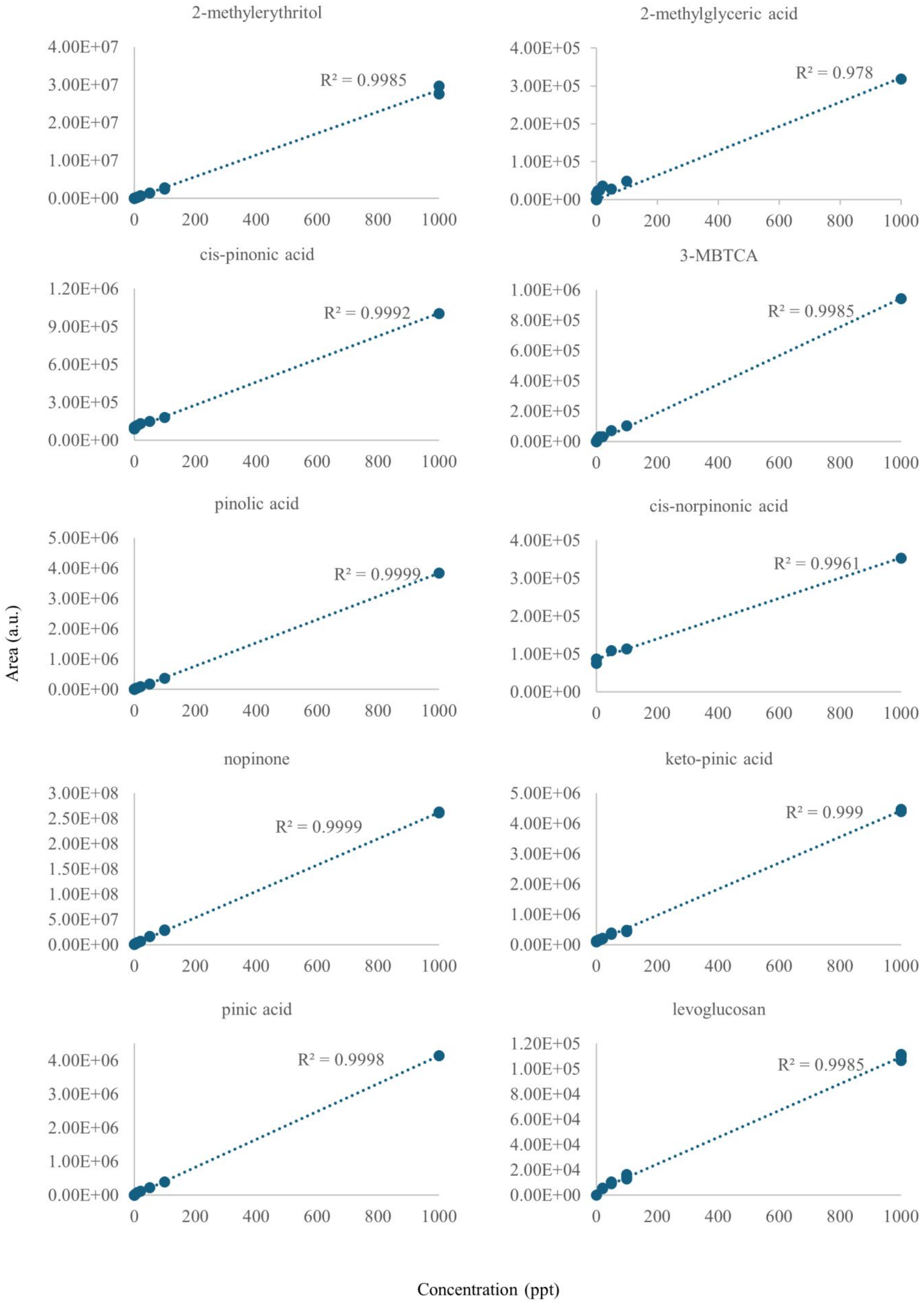

Figure S4. Full range calibration curve 1 ppt – 1 ppb for each target compound.

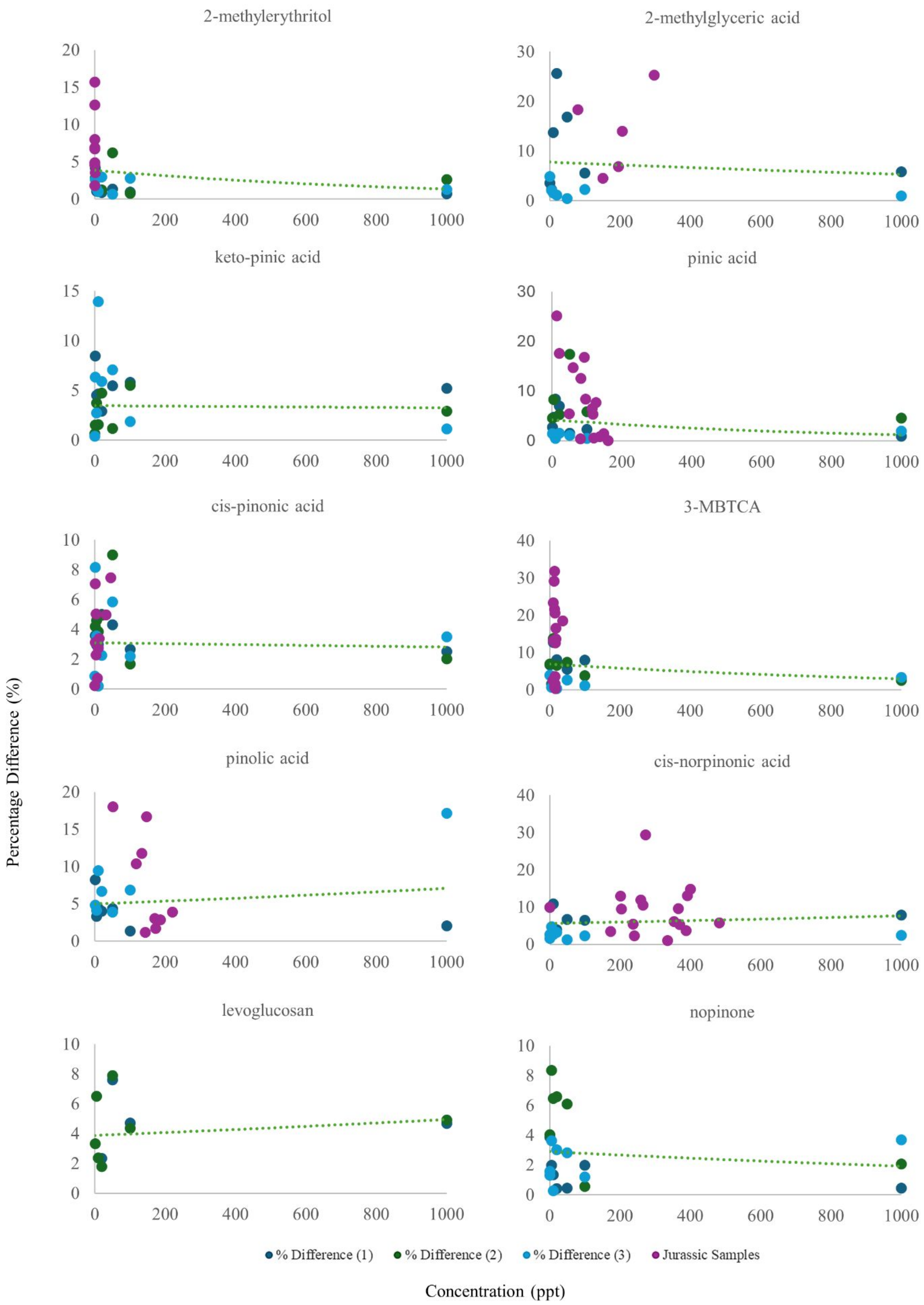

Figure S5. Exponentially fitted plots used for repeatability calculations.

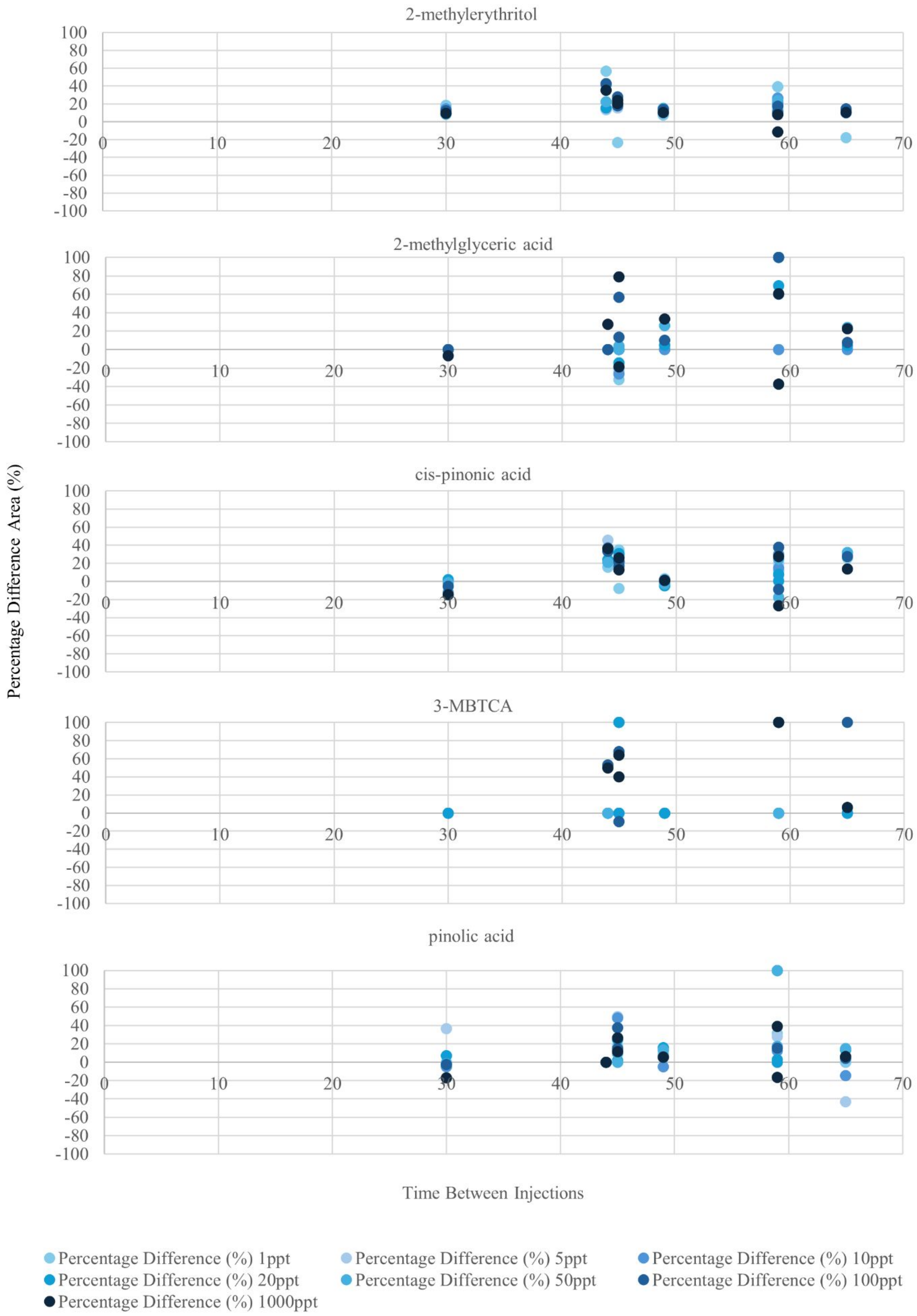

2-methylerythritol
2-methylglyceric acid
cis-pinonic acid
3-MBTCA
pinolic acid
Percentage Difference Area (%)
Time Between Injections
100
80
60
40
20
0
-20
-40
-60
-80
-100
0
10
20
30
40
50
60
70
Percentage Difference (%) 1ppt
Percentage Difference (%) 5ppt
Percentage Difference (%) 10ppt
Percentage Difference (%) 20ppt
Percentage Difference (%) 50ppt
Percentage Difference (%) 100ppt
Percentage Difference (%) 1000ppt

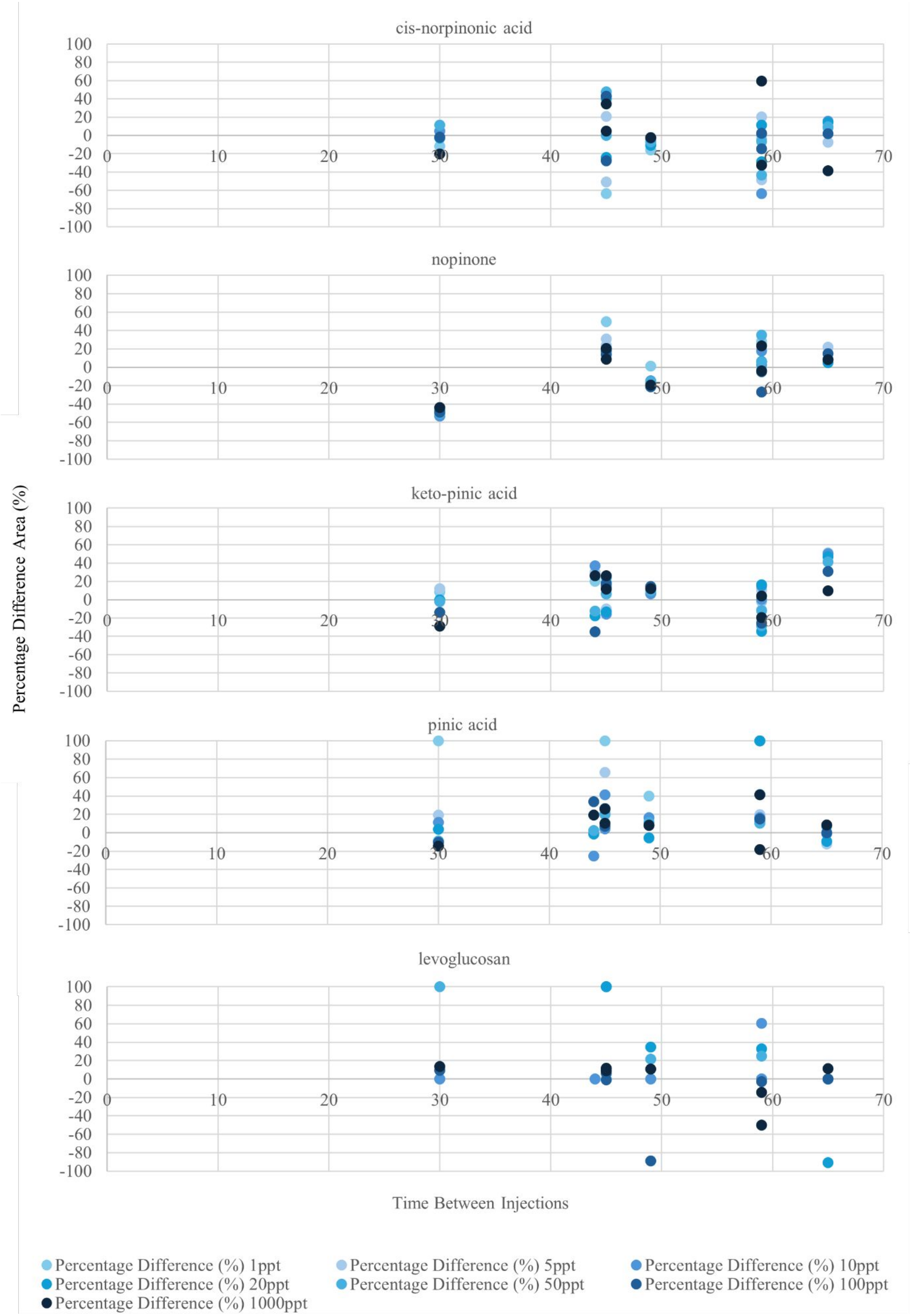

Figure S6. Plots illustrating the percentage difference in recorded area of multiple injections from the same standard vial over time. Positive values suggest degradation of compound, negative values suggest evaporation of water.

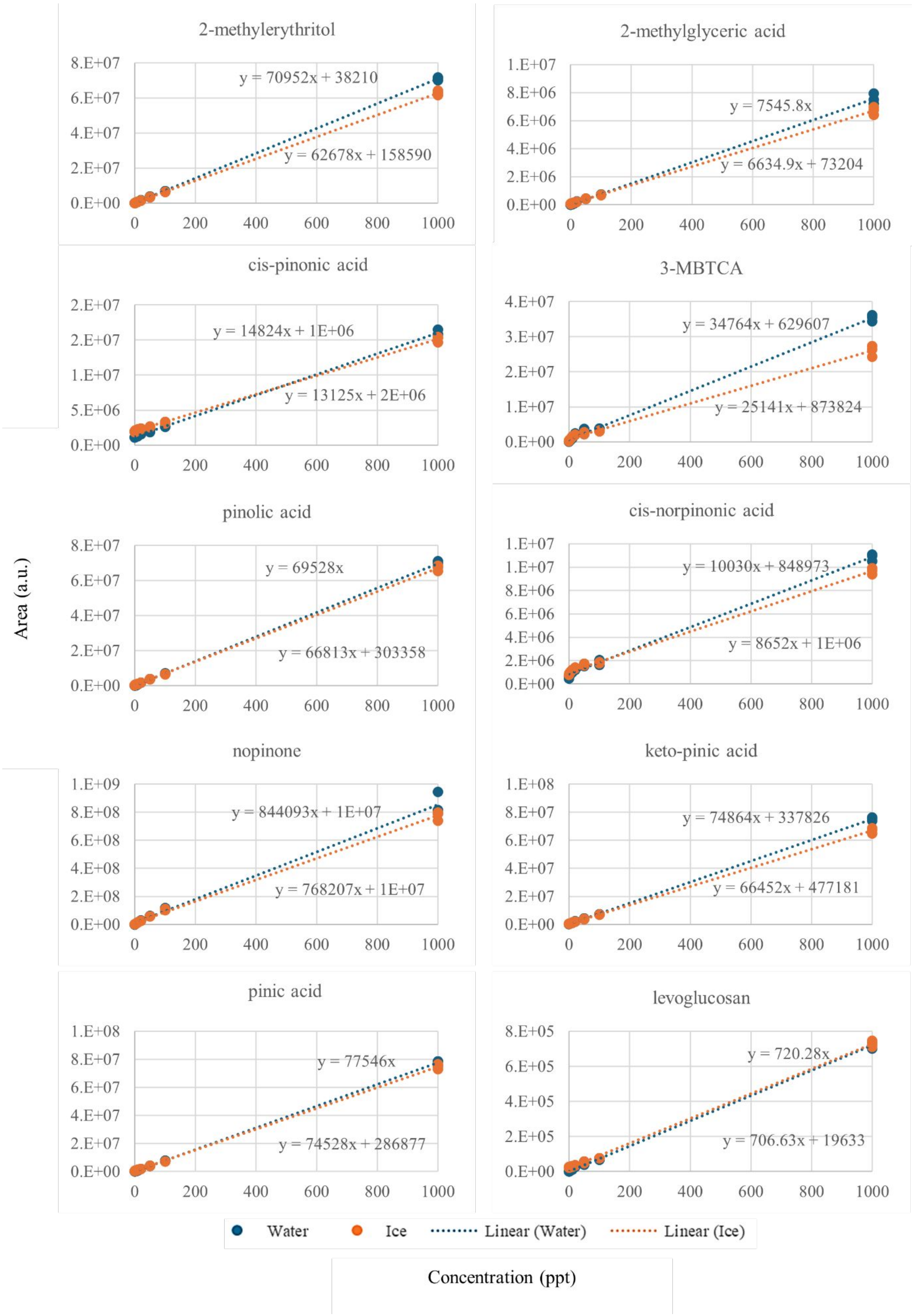

Figure S7. Plots illustrating the matrix effect of standard calibration made up in MilliQ water (blue) and melted ice core (orange) for all target compounds.

References:

Dette, H. P., Qi, M., Schröder, D. C., et al., *J. Phys. Chem.*, Glass-Forming Properties of 3-Methylbutane-1,2,3-tricarboxylic Acid and Its Mixtures with Water and Pinonic Acid, **2014**, 118, 7024-7033, doi:10.1021/jp505910w.
